# Draft Genome Sequence of *Lactobacillus gorillae* Strain KZ01^T^, Isolated from a Western Lowland Gorilla

**DOI:** 10.1128/genomeA.01196-15

**Published:** 2015-10-15

**Authors:** Sayaka Tsuchida, Maiko Nezuo, Masatoshi Tsukahara, Yoshitoshi Ogura, Tetsuya Hayashi, Kazunari Ushida

**Affiliations:** a Graduate School of Life and Environmental Sciences, Kyoto Prefectural University, Kyoto-shi, Kyoto, Japan; b BioJet Co., Ltd., Uruma-shi, Okinawa, Japan; c Department of Bacteriology, Faculty of Medical Sciences, Kyushu University, Fukuoka-shi, Fukuoka, Japan

## Abstract

Here, we report the draft genome sequence of *Lactobacillus gorillae* strain KZ01^T^ isolated from a western lowland gorilla (*Gorilla gorilla gorilla*). This genome sequence will be helpful for the comparative genomics between human and nonhuman primate-associated *Lactobacillus*.

## GENOME ANNOUNCEMENT

*Lactobacillus gorillae* bacteria were isolated from Gabonese wild and Japanese captive western lowland gorillas (1). The 16S rRNA sequence of this species was detected in the feces of wild Ugandan eastern gorillas too. *L. gorillae* is phylogenetically related to human-associated *L. fermentum*, but *L. gorillae* has not been found in humans so far. Therefore, this species can be considered as gorilla-specific or nonhuman primate specific *Lactobacillus*. Thus, the genome information of *L. gorillae* will help identify the factors involved in the adaptation of lactobacilli to the intestinal environment of the *Homininae*.

The genomic DNA of strain KZ01^T^ (=JCM19575^T^) was extracted and purified from the cells grown on a de Man, Rogosa, Sharpe (MRS) agar plate (Difco) by Quickgene Mini 80 (Kurabo, Tokyo Japan). Illumina Miseq produced 1,183,050 pair-ended reads representing an average coverage of 118-fold. The reads were assembled by CLC Genomics Workbench 8.0.1 after trimming low-quality sequences. The final draft genome consists of 63 scaffolds (from 1.1 to 106 kb in length). The total length of the genome assembly is 1,641,621 bp, with an *N*_50_ equal to 44 kbp. Gene identification and annotation was performed using the Microbial Genome Annotation Pipeline online server, and additional database searches for RefSeq, TrEMBL, clusters of orthologous groups (COGs), and KEGG (2, 3). Genes for antibiotic resistance were estimated by ARDB (4).

The genome size of *L. gorillae* strain KZ01 (1.64 Mb) is smaller than previously sequenced *Lactobacillus* species (1.8 to 3.3 Mb) (5). The G+C content of KZ01 (48.1%) is relatively higher among sequenced *Lactobacillus* species (34 to 51%) (5). The draft genome contains 53 tRNAs and 3 rRNAs (one 16S rRNA, one 23S rRNA, and one 5S rRNA) in addition to 1,582 protein coding sequences (CDSs). Among these, specific COGs were assigned to 1,439 CDSs. Genes for carbohydrate metabolism showed the highest prevalence (10.9%) followed by nucleic acid metabolism (8.4%) and amino acid metabolism (6.0%). Since antibiotic resistance of *Lactobacillus* has been issued for the safety of their probiotic use (6), we have particular interest in resistance-related genes of KZ01. KEGG and ARDB suggested the presence of genes for penicillin binding protein 1A and 2A, and the *mraY*, *murG*, and *murF* genes and those involved in the *van* operon in the KZ01 genome. In addition to these genes for the natural resistance, the presences of *catB1*, *vatB*, *macB*, and *lmrB* were suggested. The genome of *L. gorillae* KZ01 will provide a foundation for gastrointestinal microbiota of wild animals and fermentation and comparative genomics between human and nonhuman primate-associated lactobacilli.

### Nucleotide sequence accession numbers.

The sequence information was submitted to DDBJ/EMBL/GenBank under the accession no. BCAH01000001 to BCAH01000063.
